# Supplementary material for: Whole-Cell Display of Phospholipase D in Escherichia coli for High-Efficiency Extracellular Phosphatidylserine Production
Source: Biomolecules. 2024 Apr 2;14(4):430. doi: 10.3390/biom14040430 (PMC11048313; doi:10.3390/biom14040430)
Supplement: Supplementary file 1 [file biomolecules-14-00430-s001.zip › biomolecules--supplementary.pdf]

Supplementary materials

Table S1. Primers used in this study.

Gene	Primer	Sequence(5'-3')
sfGFP	S1	CATCATCACTCAGGATCGATGGTGAGCAAGGGCGAGGAGCTGTTCAC
	S2	ACGGAAGCTGATGATCATTGGACCCTGGAACAAAACCTCCAATTTATA
PLDr34	P1	ATGATCATCAGCTTCCGTCTGAGCCGTCCG
	P2	TAGCAGCCGGATCTCAAATATGACACAGGCCACGTTCA
pET-28a	A1	TGAGATCCGGCTGCTAACAAAGCCCGAAAGGAAGCT
	A2	CGATCCTGAGTGATGATGATGATGATGGCTGCTGCCCA
pET-23a	A3	TGAGATCCGGCTGCTAACAAAGCCCGAAAGGAAGCTGA
	A4	CGATCCTGAGTGATGATGATGATGATGCATATGTATATCT

Table S2. Plasmid and strains used in this study.

Strain/Plasmid	Description	Reference
pET-28a	Vector for expression proteins,T7 promoter,Kan^r	This study
pET-23a	Vector for expression proteins,T7 promoter,Amp^r	This study
pPLDr34	pET-28a encoding PLDr34, Kan^r	This study
psfGFP-PLDr34	pET-28a/ pET-23a encoding sfGFP-PLDr34, Kan^r/Amp^r	This study
Strain		
E-PLDr34	*E.coli* Rosetta Blue (DE3) (pPLDr34)	This study
E-sfGFP-PLDr34	*E.coli* Rosetta Blue (DE3) (psfGFP-PLDr34)	This study

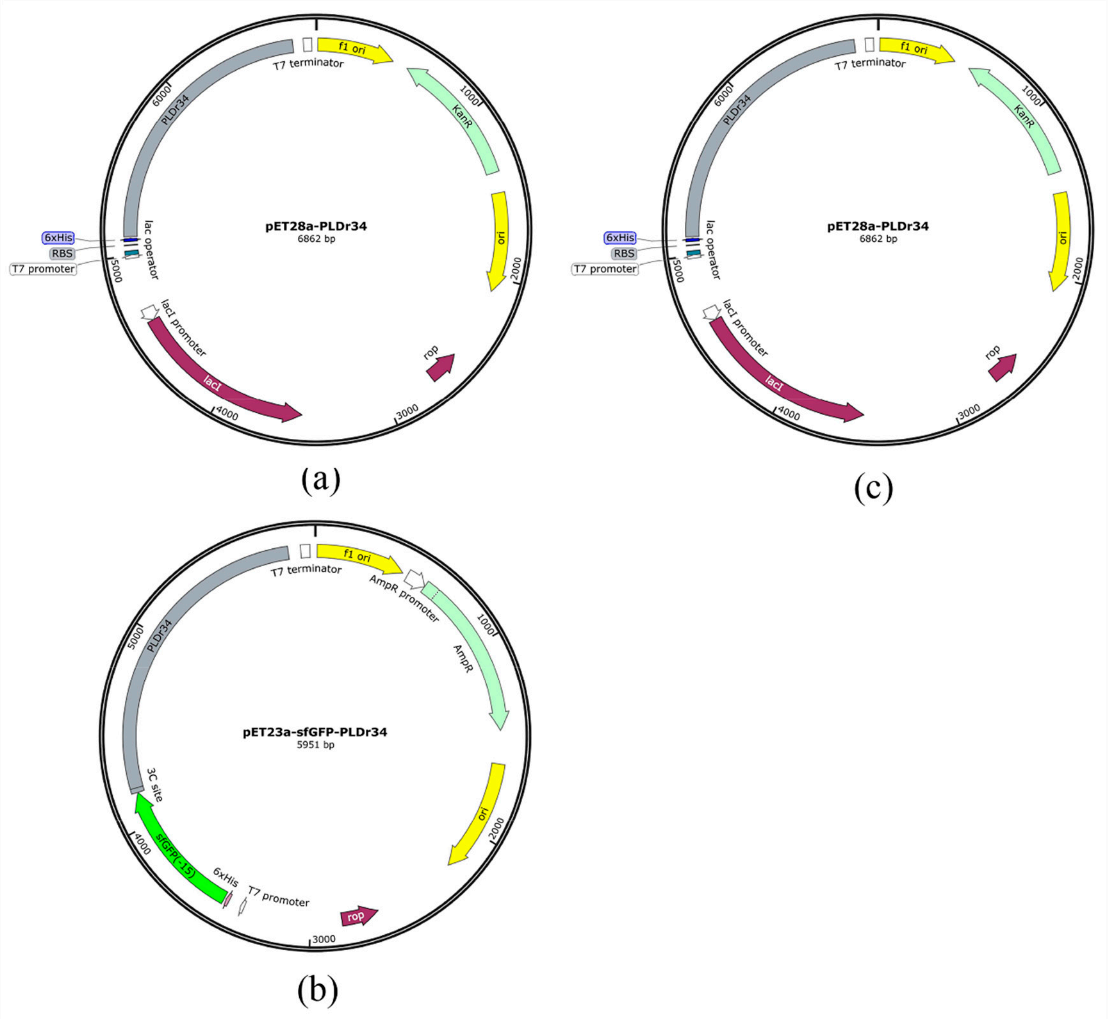

Figure S1. Three expression plasmid maps have been constructed. (a) pET28a-PLDr34. (b) pET23a-sfGFP-PLDr34. (c) pET28a-sfGFP-PLDr34.

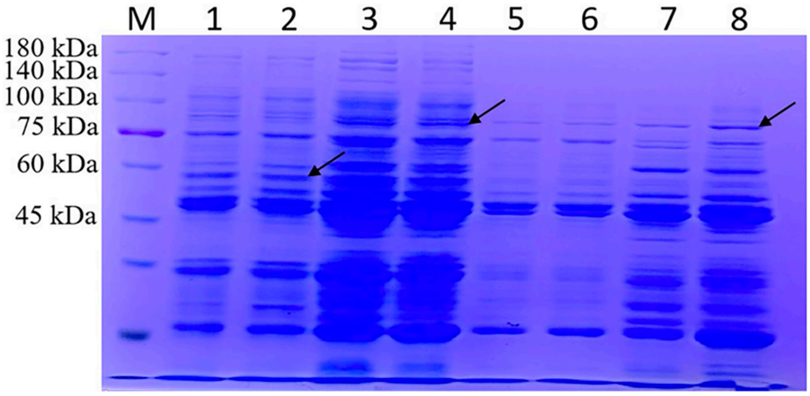

Figure S2. SDS-PAGE analysis of whole cells and Washing membrane protein. M : protein marker; lane 1: the total cell of pET28a-PLDr34 before induction; lane 2: the total cell of pET28a-PLDr34 after induction; lane 3: the total cell of pET28a-sfGFP-PLDr34 before induction; lane 4: the total cell of pET28a-sfGFP-PLDr34 after induction;lane 5:Washing membrane protein of pET28a-PLDr34 before induction; lane 6: Washing membrane protein of pET28a-PLDr34 after induction; lane 7: Washing membrane protein of pET28a-sfGFP-PLDr34 before induction; lane 8: Washing membrane protein of pET28a-sfGFP-PLDr34 after induction.

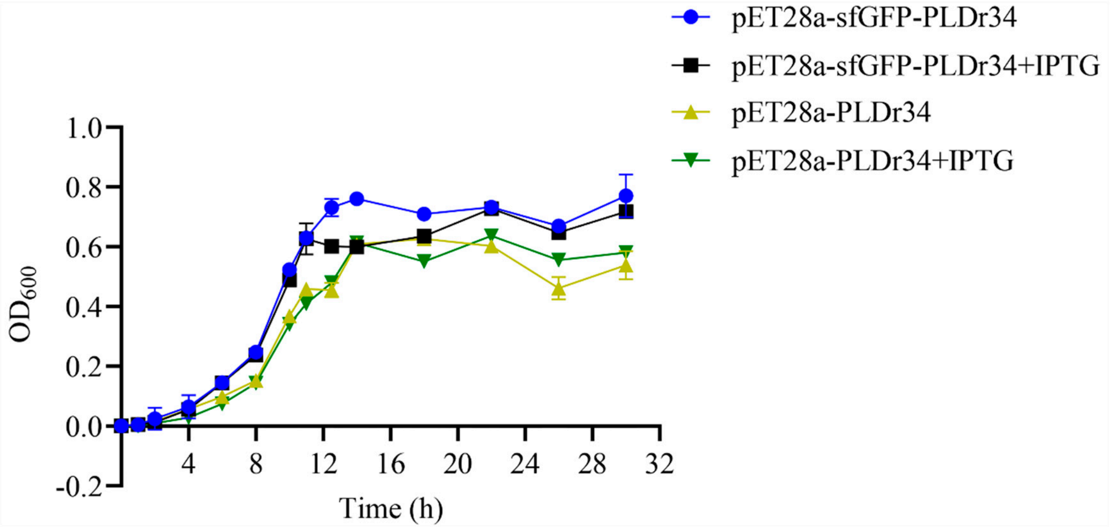

Figure S3 . The growth curves of pET28a-PLDr34 and pET28a-sfGFP-PLDr34 in LB medium.

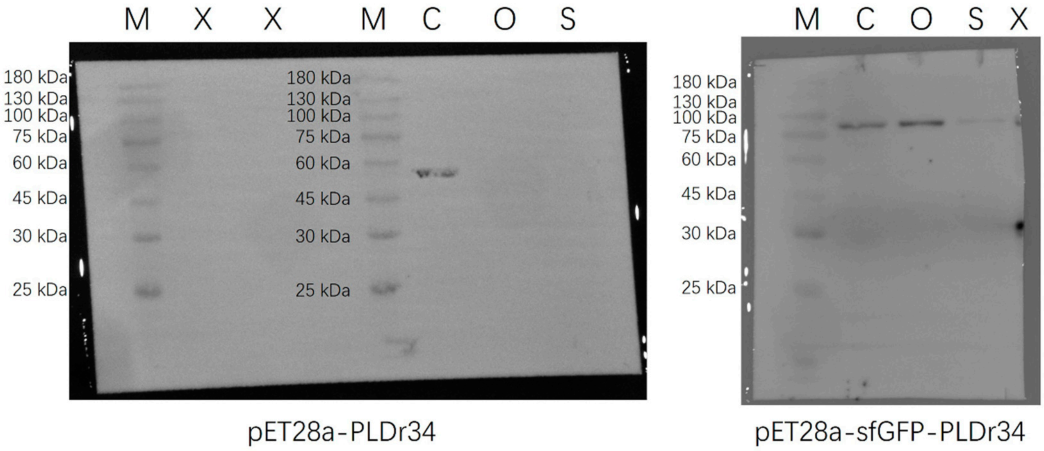

Figure S4. The original images of Figure 1b. M: protein marker; C: the Whole cell; O: the outer membrane; S: the medium supernatant; X: the other samples.
