# Supplementary material for: Whole-Cell Display of Phospholipase D in Escherichia coli for High-Efficiency Extracellular Phosphatidylserine Production
Source: Biomolecules. 2024 Apr 2;14(4):430. doi: 10.3390/biom14040430 (PMC11048313; doi:10.3390/biom14040430)
Supplement: Supplementary file 1 [file biomolecules-14-00430-s001.zip › biomolecules-2881088-original-images.pdf]

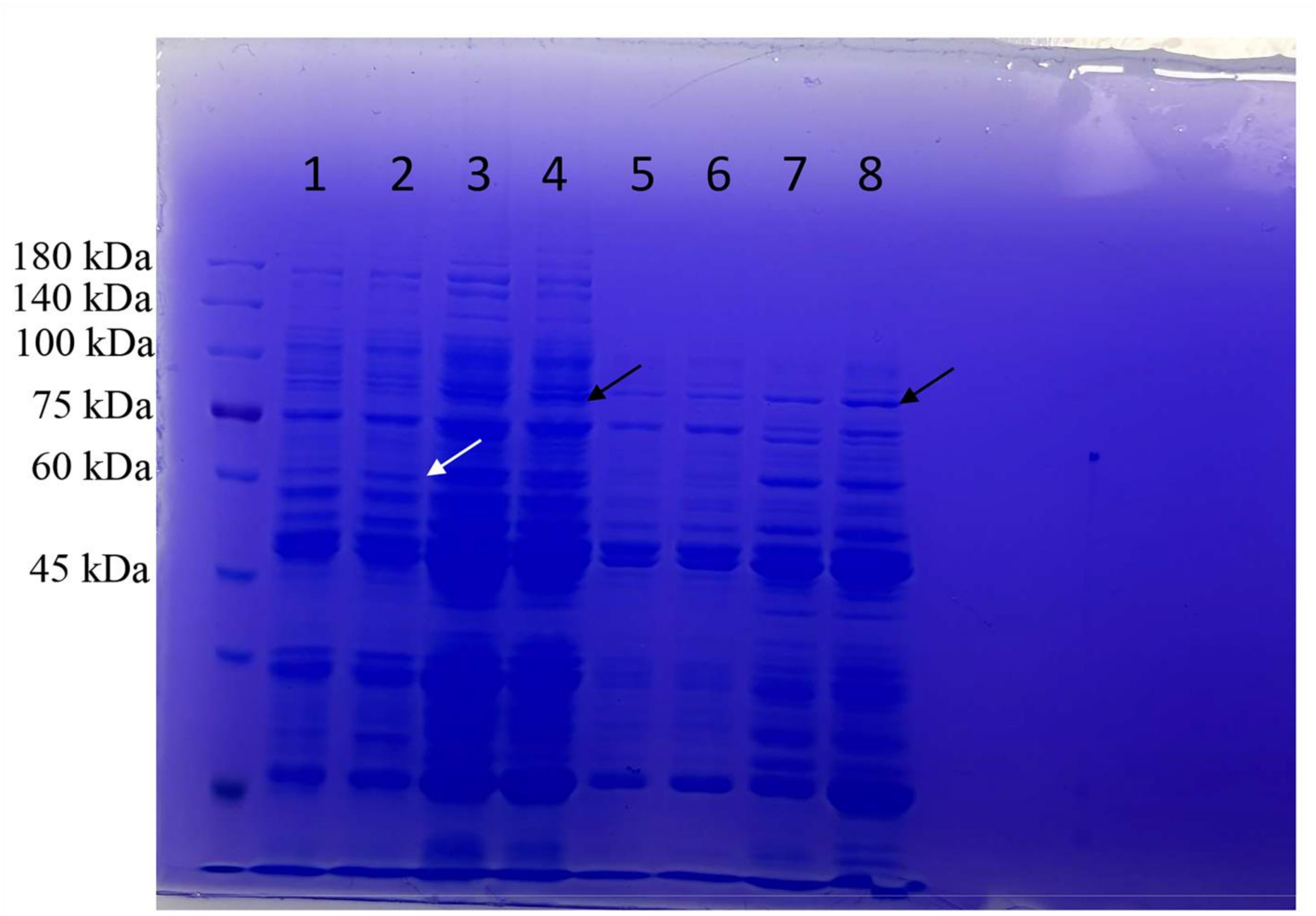

Figure 1.(b) SDS-PAGE analysis of whole cells and Washing membrane protein. The molecular weight marker is on the left. M : protein marker; lane 1: the total cell of pET28a-PLDr34 before induction; lane 2: the total cell of pET28a-PLDr34 after induction; lane 3: the total cell of pET28a-sfGFP-PLDr34 before induction; lane 4: the total cell of pET28a-sfGFP-PLDr34 after induction; lane 5: Washing membrane protein of pET28a-PLDr34 before induction;lane 6: Washing membrane protein of pET28a-PLDr34 after induction; lane 7: Washing membrane protein of pET28a-sfGFP-PLDr34 before induction; lane 8: Washing membrane protein of pET28a-sfGFP-PLDr34 after induction.
